# Cardiovascular disease-related miRNAs expression: potential role as biomarkers and effects of training exercise

**DOI:** 10.18632/oncotarget.24428

**Published:** 2018-03-30

**Authors:** Simona Ultimo, Giorgio Zauli, Alberto M. Martelli, Marco Vitale, James A. McCubrey, Silvano Capitani, Luca M. Neri

**Affiliations:** ^1^ Department of Morphology, Surgery and Experimental Medicine, University of Ferrara, Ferrara, Italy; ^2^ Department of Biomedical and Neuromotor Sciences, University of Bologna, Bologna, Italy; ^3^ Department of Medicine and Surgery, University of Parma, Parma, Italy; ^4^ CoreLab, Azienda Ospedaliero, Universitaria di Parma, Parma, Italy; ^5^ Department of Microbiology and Immunology, Brody School of Medicine, East Carolina University, Greenville, NC, USA

**Keywords:** cardiovascular diseases, sport exercise, aging, miRNAs, biomarkers

## Abstract

Cardiovascular diseases (CVDs) are one of the most important causes of mortality worldwide, therefore the need of effective preventive strategies is imperative. Aging is associated with significant changes in both cardiovascular structure and function that lower the threshold for clinical signs and symptoms, making older people more susceptible to CVDs morbidity and mortality.

microRNAs (miRNAs) modulate gene expression at post-transcriptional level and increasing evidence has shown that miRNAs are involved in cardiovascular physiology and in the pathogenesis of CVDs.

Physical activity is recommended by the medical community and the cardiovascular benefits of exercise are multifactorial and include important systemic effects on skeletal muscle, the peripheral vasculature, metabolism, and neuroendocrine systems, as well as beneficial modifications within the myocardium itself.

In this review we describe the role of miRNAs and their dysregulation in several types of CVDs. We provide an overview of miRNAs in CVDs and of the effects of physical activity on miRNA regulation involved in both cardiovascular pathologies and age-related cardiovascular changes and diseases.

Circulating miRNAs in response to acute and chronic sport exercise appear to be modulated following training exercise, and may furthermore serve as potential biomarkers for CVDs and different age-related CVDs.

## INTRODUCTION

According to the report of Centers for Disease Control and Prevention and the National Health and Nutrition Examination Survey III in US, about 47% of deaths is due to cardiovascular diseases (CVDs) [1]. CVDs include several diseases such as hypertension, hyperlipidemia, coronary artery disease (CAD), congestive heart failure (CHF), stroke, cardiac hypertrophy (CH) and arrhythmia. Inactivity or a sedentary lifestyle is associated with increased cardiovascular events and premature death [2].

The average lifespan of the human population in so called developed countries is increasing worldwide, mostly because of declining fertility and increasing longevity. It has been predicted that, in 2035, nearly one in four individuals will be 65 years or older [3] and the prevalence of CVDs increases dramatically in parallel with age i.e. there is a clear association between aging of the population and increasing incidence of CVD [4]. The prevalence of heart failure (HF) in the adult population in developed countries is 1–2%, which rises to >10% among persons 70 years or older [5].

Regular physical activity reduces several cardiovascular risk such as hypertension [6, 7] and diabetes mellitus [8]. Increased levels of physical activity and exercise are not only associated with a significant reduction in cardiovascular mortality and morbidity in later life [9, 10], but also have substantial impact on the “rate of aging” (healthspan) in the absence of disease [11].

Aging is associated with a significant decrease in physical activity and an increase in sedentary behavior (sitting time) that taken together further increase the risk of cardiovascular mortality and morbidity [12].

Evidence from many scientific studies shows that reducing these risk factors in parallel decreases the chance of heart attack or another cardiovascular event, such as a stroke and reduces the need of a coronary revascularization procedure [13]. Exercise promotes weight reduction and help lowering blood pressure. Exercise can reduce “bad” cholesterol levels in the blood (the low-density lipoprotein [LDL] level), as well as total cholesterol and can raise the “good” cholesterol (the high-density lipoprotein level [HDL]) [14]. The effect of permanent physical exercise on overall cardiovascular risk, when combined with other lifestyle modifications (such as proper nutrition, smoking cessation and appropriate medication use), can be fundamental [15] since physical fitness and level of regular exercise are closely related to cardiovascular health.

Large-scale epidemiological studies performed in healthy subjects and in patients with CVD demonstrated that low aerobic exercise capacity was a stronger predictor of mortality than other established risk factors [16–18]. Conversely, high physical activity levels and cardiorespiratory fitness were associated with low risk of CVD and mortality [16, 19]. It has also been demonstrated that cardiorespiratory fitness, measured as maximal oxygen uptake (VO_2 max_), is a good indicator of cardiovascular health [20, 21]. Levels of physical activity capable to increase cardiorespiratory fitness may therefore be one of the most important modifiable life-style interventions indicated for preventing or controlling cardiovascular, metabolic and skeletal muscle diseases [22, 23]. In the heart, a pivotal feature of regular exercise training is improved systolic and diastolic function and larger cardiac output [24, 25]. The long term effects of exercise training includes cardiomyocyte shortening and rates of contraction-relaxation improvements, providing a cellular basis for a better organ function [26].

In contrast, CVD in general induces cardiac dysfunction with reduced ejection fraction and lower cardiac output, associated also with impaired cardiomyocyte contractile function [27]. Experimental studies suggested restored contractility and attenuated pathological growth as important cellular mechanisms for the beneficial effects of physical activity in HF [26, 28].

The identification of specific miRNAs has opened up a new field of investigation to understand the molecular mechanisms controlling gene expression also in cardiovascular development and diseases [29, 30].

In this review, we report an overview about the role and involvement of miRNAs in several CVDs and their implications for functional capacity in age-related CVDs.

We also highlight the benefits of exercise training on CVDs, the long-term effects of diverse exercise training modalities (e.g. running, cycling, resistance training) in the cardiac miRNA profile are properly addressed, including circulating miRNA profiles in aging-related CVDs suggesting their potential role as new therapeutic biomarkers in the cardiovascular field.

miRNAs play pivotal roles in CVDs [31].

Several studies that postulate the relations between CH, hypertension and miRNAs have emerged. The miRNAs more frequently cited in cardiomyocytes studies are the miR-1, -133, -30, -21, -98, -378, -221, -22, -27, -212/132, -199 and -350 with several targets that are involved in the adaptive response of CH [32].

Changes in cardiac miRNA expression levels have been also associated with cardiac stress and development of cardiac hypertrophy due to pressure overload [33–35], myocardial infarction (MI) [36, 37] and also, in humans, end-stage HF [38–41].

We have focused on the description of main miRNAs involved in hypertension, hyperlipidemia, CAD, CHF, stroke, arrhythmias and CH.

### miRNA expression in Hypertension

Several researches showed that miRNAs directly or indirectly influence hypertension. Sixty hypertensive patients and 29 healthy individuals were studied for assessment of miR-9 and miR-126 levels: both miRNAs were lowered in serum samples of hypertensive patients when compared to healthy control [42, 43].

Another study, found that 46 miRNAs were upregulated in serum samples of hypertensive patients compared with healthy individuals, among which 9 miRNAs were upregulated (i.e. human cytomegalovirus-miR-UL112, miR-605, miR-623, miR-let-7e, miR-516b, miR-600, kshv-miR-K12-6-3p, miR-602 and miR-1252). Eighteen miRNAs were downregulated, such as for example miR-296-5p, miR-133b, miR-625,miR-let-7a and miR-206 [44], miR-1236 and miR-518b [42].

In a study it has been found that physical exercise may alter the expression of specific miRNAs targeting renin–angiotensin–aldosterone system (RAAS) genes. The RAAS system has a pivotal role in the pathogenesis of hypertension [45, 46]. Its persistent and pathological activation can lead to alteration in blood pressure, cardiac contractility, electrolyte balance, as well as vascular resistance and tone [47]. Several studies indicate miRNAs, such as miR-206 and miR-126 (Table 1), to be intrinsically connected with all the components of this hormonal system [46]. Furthermore they demonstrated in the same study that decrease in miR-143 expression enhanced cardioprotective genes (such as NF-KB and apolipoprotein D (Apo D)) and decrease miR-27 which in heart is an inhibitor of angiotensin converting enzymes (ACE) [48]. The results imply that a decrease in miR-143 could upregulate cardioprotective genes in the heart and an increase of miR-27 expression inhibits ACE levels. These results suggest that a basis for treatment to prevent the development of pathological left ventricular hypertrophy (LVH) might be the inhibition of specific miRNAs, with antisense miRNA or siRNA [48].

**Table 1:** Summarized list of the most studied miRNAs in the described CVDs

miRNA	MAIN TARGET	GENOMIC LOCALIZATION	CVDs	MECHANISM OF ACTION	REFERENCES
miR-21	EPCs, ERK/MAP	chr17:59,841,266-59,841,337	CAD, CHF, Stroke	Cardiac remodelling	Fleissner F *et al.*, 2010; Ali SS. *et al.*, 2015
miR-27a	ACE, CDK5	chr19:13,836,440-13,836,517	Hypertension, Stroke,	Prediction of LV remodelling	Sepramaniam S *et al.*, 2014; Ali SS. *et al.*, 2015
miR-126	RAAS, VCAM-1	chr9:136,670,602-136,670,686	Hypertension, CHF, Stroke	Regulation vascular integrity and angiogenesis	Kontaraki JE *et al.*, 2014; Ali SS. *et al.*, 2015;
miR-133a/133b	Calcineurin, SGK1, IGFR1	chr18:21,825,698-21,825,785 chr6:52,148,923-52,149,041	Hypertension, CAD, CH	Cardiac development	Lew JK *et al.*,2017; Ali SS. *et al.*, 2015
miR-143/145	NF-KB, Apo D, ACE	chr5:149,428,918-149,429,023; chr5:149,430,646-149,430,733	Hypertension, CAD, Stroke	Macrophage differentiation and polarized activation processes	Fernandes T *et al.*, 2011; Ali SS. *et al.*, 2015
miR-221	EPCs	chrX:45,746,157-45,746,266	CAD, Stroke	Mobilization of ECs	Jamaluddin MS *et al.*, 2011; Ali SS. *et al.*, 2015

ACE catalyzes the conversion of angiotensin I (Ang I) in its bioactive form angiotensin II (Ang II), the main effector of RAAS [46]. ACE is a target of miR-143/145 cluster (Table 1). Expression of this cluster was increased by shear stress in endothelial cells (ECs) *via* activation of adenosine monophosphate-activated protein kinase (AMPK) -p53 pathway, leading to a reduction in ACE gene expression [49]. Moreover, miR-143/145 expression was fundamental to maintain the contractile phenotype of vascular smooth muscle cells (VSMCs) *in vitro* and a regular blood pressure *in vivo*. Loss of miR-143/145 led to an ACE enzyme overexpression and a shift from contractile to synthetic (i.e. synthesis of extracellular matrix (ECM)) phenotype of VSMCs, which increased the probability to develop neointimal lesions [50]. Synthetic VSMCs are characterized by increased proliferation and migration ability. These cells sustain the vascular remodeling occurring during hypertension [51].

Some miRNAs contribute to vascular remodeling which perpetuates hypertension [52], like miR-130a, which regulates vascular smooth muscle cells contributing to vascular remodeling in hypertension [53]. During the development of pulmonary arterial hypertension it has been shown that miR-22 and miR-30 levels were decreased whereas miR-322 and miR-451 were increased in hypoxic and monocrotaline model, that are rat models of pulmonary hypertension [31]. miR-30 is known to play a role in extracellular matrix remodeling in heart and is expressed in experimental model of other kind of hypertension such as pulmonary hypertension [54].

### miRNA expression in Hyperlipidemia

A sedentary lifestyle, a nutrient poor diet and genetic predisposition often contributes to the development of altered lipid profile in the blood with resultant hyperlipidemia, an established risk factor for CVDs [55]. A regulation of miRNAs is critical for lipid homeostasis and prevention of CVDs induced by lipid overload (e.g. CAD) [55].

For example, miR-122 accounts for 70% of miRNAs in the liver, serves as a primary regulator of lipid biosynthesis and aberrant levels have been implicated in hyperlipidemia of patients with CAD [56, 57]. Pharmacological inhibition of miR-122 in mice and non-human primates [58, 59] and genetic knockout of miR-122 in mouse [60, 61], lead to a decrease in plasma cholesterol levels [62]. miR-122 plays an important role in the fatty acid metabolism: treatment with antisense oligonucleotides (ASOs) against miR-122 has been shown to prevent hepatic steatosis [59]. However, both whole body or liver specific miR-122 knockout mice were found to have enhanced susceptibility to hepatic steatosis [62]. The ability of miR-122 inhibition to lower cholesterol and reduce lipid accumulation in the liver suggests that anti-miR-122 therapy may be a promising approach for the treatment of cardiovascular and other metabolic diseases [62].

Increased levels of miR-30c have been shown to reduce plasma lipids, due to decreased cholesterol secretion by targeting microsomal triglyceride transfer protein (MTP) [62]. Overexpression of miR-30c was found to reduce plaque formation in the atherosclerosis prone apolipoprotein E (Apo E) deficient mouse model, while inhibition of miR-30c caused severe hyperlipidemia and atherosclerotic plaque formation, thus suggesting that also miR-30c mimetics may be a promising therapeutic approach in patients at risk of CVDs [63].

miR-33 embedded in the sterol regulatory element binding protein 1/2 (SREBP1/2) genes, is a regulator of cholesterol homeostasis. Several studies have demonstrated that genetic ablation of miR-33 enhances plasma HDL levels [64, 65] and promotes accumulation of atheroprotective M2 macrophages. Additional studies revealed that in non-human primates, pharmaceutical antagonism of miR-33 reduced very low density lipoprotein (VLDL) triglycerides by 50%, while plasma HDL levels increased by 40% at 12 weeks. The differential effects of miR-33 on HDL and VLDL are due to active depletion of the hepatocyte cholesterol pool by the ATP-binding cassette transporter (ABCA1) that export phospholipids and cholesterol outside cells to apolipoprotein A1 (Apo A1) yielding high levels of HDL. Therefore, cholesterol depletion resulted in attenuated VLDL secretion [66].

Overall these studies reveal that miRNAs are integrated in the complex genetic networks that regulate cholesterol homeostasis [62].

### miRNA expression in CAD

Changes in cardiac miRNA expression levels have also been described in CVD patients. A few years ago, a randomized clinical trial study was conducted by Slagvold *et al.* (2014) demonstrating that remote ischemic preconditioning (RIPC) preserves mitochondrial function and influences miRNA expression in atrial myocardium during coronary bypass surgery [67].

Several studies demonstrated that reduction of endothelial expressed miRNAs may be linked to atherosclerotic lesions within the vasculature of CAD patients [31, 54, 68, 69]. Smooth muscle-enriched miR-145 are reduced in patients with CAD whereas cardiac muscle-enriched miRNAs, miR-133 and miR-208a, are further increased in patients with CAD [69]. Patients with CAD showed increased levels of miR-221 (Table 1) and miR-222 in endothelial progenitor cells (EPCs), and these miRNAs lead to mobilization of EPCs [31, 54, 68, 69]. Apart from direct regulating condition of CAD, miRNAs are involved indirectly as well, since it has been shown that oxidative stress defences in human EPCs are regulated by miR-21 (Table 1) [70].

### miRNA expression in CHF

Goren *et al.* (2012) screened 186 miRNAs in serum of CHF patients. Four of them i.e. miR-423-5p, miR-320a, miR-22 and miR-92b, were found to be increased in serum of HF patients and a significant association was revealed between the same miRNAs and prognostic parameters like elevated serum brain natriuretic peptide level and dilatation of the left ventricle and atrium [71].

Plasma from 12 CHF patients were compared with 12 healthy subjects by using microarray method, which revealed expression of 108 miRNAs in CHF patients. Among these 108 miRNAs, miR-423-5p and miR-126 were highly expressed in CHF. Decreased levels of miR-107, miR-139 and miR-142-5p were also displayed in HF conditions. Cakmak *et al.* [72] have demonstrated the relationship between expression of miRNAs and electrocardiogram parameters related to left ventricular mass index (LVMI) in patients with CHF [43]. Results in this study demonstrated that 29 miRNAs were altered in CHF patients. Among these 29 miRNAs,miR-182, miR-200a-star and miR-568, were found to have inverse correlation with LVMI, whereas two miRNAs i.e. miR-155 and miR-595 were found to have direct correlation with LVMI [43, 73].

By using microarray profiling method in CHF patients it has been shown upregulation of 18 miRNAs: i.e. miR-21, miR-4278, miR-650, miR744star and miR-516-5p, and downregulation of 11 miRNAs: i.e. miR-129-3p, miR-3155, miR-3175 and miR-583 [43, 73].

Identify these miRNAs was very significant in terms of clinical diagnosis suggesting that they could be used as potential biomarkers in CHF [74].

To explore the role of miRNAs in pathological cardiac growth and in HF, three miRNAs were studied by using transgenic mice. These studies have shown that miR-195 and miR-100 were upregulated whereas miR-92 was downregulated [75] suggesting that different level of miRNAs can play a role in HF.

### miRNA expression in stroke

Stroke is responsible for 10% of deaths worldwide and is one of the leading causes of disability and miRNAs have to be included among stroke risk factors including also hypertension, atherosclerosis, atrial fibrillation, diabetes and dyslipidemia [43, 76].

miRNAs like miR-21, miR-221 and miR-145 are known to be associated with the cardiovascular system (Table 1). On this basis, their levels were evaluated in serum samples of patients with stroke. Results in this study demonstrated that miR-21 and miR-222 were novel biomarkers of stroke but miR-145 were not [77].

By real-time PCR technique, levels of miRNAs were quantified in serum samples of 197 patients with ischemic stroke at interval of 24 h, of 1, 4 and 48 weeks. Fifty healthy volunteers were selected as control. Results showed that circulating levels of miR-301 and miR-126 were downregulated whereas let-7b was upregulated in ischemic stroke patients up to 24 weeks. However, levels of these miRNAs were normalized 48 weeks after the symptom onset. The researchers concluded that miR-30a, miR-126 and let-7b can be employed as biomarkers of ischemic stroke [78].

Atherosclerosis is the major cause of ischemic stroke, either in carotid or intracranial artery [79]. The foam cells in the plaque are derived from the macrophages absorbing the oxidized LDL in which miRNAs play a role. In particular miR-126 could target the 3′ untranslated region (UTR) of vascular cell adhesion molecule 1 (VCAM-1) and modulate its expression (Table 1) [80]. The VCAM-1 is expressed in the activated endothelial cells to promote the adhesion of macrophages to vascular endothelium which plays a role in the development of atherosclerosis [81].

Sepramaniam *et al.* [82] studied a panel of 32 miRNAs blood miRNAs, among which a consistent upregulation of miR-125b-2, miR-27a, miR-422a, miR-488 and miR-627 was found during ischemic stroke. Thus, it was concluded that these miRNAs possess a diagnostic value and reflects the onset of ischemic stroke. miR-27a is a direct target of cyclin-dependent kinase 5 (CDK5), which is predominantly expressed in the central nervous system (Table 1) [83]. CDK5 protein is upregulated in acute ischemia in various stroke models [84] and plays crucial roles in neuronal survival and death. Thus upregulation of miR-27a expression upon acute ischemia could be a defense mechanism by the cells to control the translation of CDK5 molecules [82].

In a study, plasma levels of miR-107 and glutamate were elevated proportionally in patients with ischemic stroke. Hence, also miR-107 plasma level may act as a biomarker for monitoring citotoxicity in ischemic stroke patients [85].

### miRNAs expression in Arrhythmias

Atrial fibrillation (AF) is the most common arrhythmia [86], especially among elderly populations, and is the end-stage manifestation of multiple pathological changes, including both structural and electrical remodeling [87]. A study demonstrated that miR-1 levels were markedly reduced (≈86%) in atrial tissue of patients with AF and a possible effect of miR-1 on in ward-rectifier K^+^ currents (I_K1_) was postulated [88]. Lu *et al.* [89] reported a 3.5-fold elevation of miR-328 levels in atrial samples of AF patients and furthermore demonstrated that the overexpression of miR-328 enhanced AF vulnerability and on the contrary knockdown of miR-328 reduced AF vulnerability in mouse models. miR-223 and miR-664 were also elevated, miR-1 was unaltered. Despite this, miR-1 has also been implicated in the modulation of a wide variety of Ca^2+^ handling proteins [87] and has been linked to ventricular arrhythmias [65].

### miRNAs expression in Cardiac Hypertrophy

Cardiac Hypertrophy (CH) is an important compensatory mechanism of the heart in response to diverse pathophysiological stimuli and the involvement of miRNAs in this pathological process is now recognized [90].

A number of miRNAs are found to be involved in CH in particular miR-1 and miR-133. Embryonic overexpression of miR-1 *in vivo* results in thin-walled ventricles, whereas miR-1 knockout mice display chambers with thickened walls [91]. miR-1 is down-regulated at the onset of pressure overload on the heart at the beginning and progression of CH [92]. The cytoskeleton regulatory protein, twinfilin-1, is a target of miR-1 and reduction of miR-1 by hypertrophic stimuli up-regulates twinfilin-1 which evokes hypertrophy through regulation of the cardiac cytoskeleton [44].

miR-133 has a critical role in determining cardiomyocyte hypertrophy since its overexpression inhibits hypertrophy whereas its suppression induces hypertrophy both *in vitro* and *in vivo* [93]. In both *in vivo* and *in vitro* models of CH miR-133 expression is down-regulated whereas calcineurin activity is enhanced [94]. In addition, these authors found that inhibition of calcineurin by cyclosporine A prevented the down-regulation of miR-133 in CH. These results indicate that miR-133 and calcineurin are reciprocally repressed in CH. Moreover, another study indicated that miR-133a plays a role in diabetes-induced cardiomyocyte hypertrophy; miR-133a down-regulation alters gene expression and mediates glucose-induced cardiomyocyte hypertrophy through Serine/threonine-protein kinase 1 (SGK1) and the insulin-like growth factor 1 (IGFR1) (Table 1).

Care and colleagues assessed the role of cardiac miRNAs in the three murine models of CH: in all three models (pathological or physiological cardiac hypertrophy) both human miR-133 and miR-1 resulted in a reduced expression [95].

## SENESCENCE OF HEART AND VASCULAR TISSUE

Aging is associated with a progressive decline in several physiological processes, leading to an increased risk of health complications and diseases. Aging has a remarkable effect on heart and arterial system, increases CVDs including atherosclerosis, hypertension, MI and stroke [4].

Aging of cardiovascular tissues are exemplified by pathological alterations including hypertrophy, altered left ventricular (LV) diastolic function and diminished LV systolic reverse capacity, increased arterial stiffness and impaired endothelial function [3]. However, the health of the arterial and cardiac systems is not mutually exclusive, as each system greatly affects the other [4].

Heart rate is influenced not only by the loss of cells in the sinoatrial node (responsible for controlling heart rate) but also by structural changes in the heart, including fibrosis and hypertrophy, which slow the propagation of electric impulse throughout the heart [96].

A reduction in cardiac output due to functional decline with age stimulates the myocardium to activate compensation mechanisms by increasing muscle mass undergoing cardiac hypertrophy; however this may provide short-term enhancement of cardiac output, whereas the long-term effect of hypertrophy diminishes cardiac function [97].

Aging of the vasculature results in increased arterial thickening and stiffness as well as dysfunctional endothelium. Clinically, these changes result in increased systolic pressure and display major risk factors for development of atherosclerosis, hypertension, stroke and arterial fibrillation [97]. Vascular dysfunction associated with aging leads to a variety of age-related pathologies, including loss of adequate tissue perfusion (resulting in ischemia), insufficient vascular growth or regression (resulting in hypertension).

The heart undergoes complex changes during aging that result in affecting the cellular composition, marked by a decrease in absolute number of cardiomyocytes that is due to increased apoptosis and necrosis and a decrease in repopulation of cardiomyocytes from cardiac stem cell reserves [98]. With age, cardiomyocytes become more susceptible to oxidative stress [99]. Measuring cardiac-specific senescence, DNA damage, as well as levels of apoptosis, necrosis, and fibrosis in animal models of aging, will lead to a better understanding of the link between aging and CVD.

### miRNA in cardiovascular aging and age-related CVDs

A number of miRNAs have been described to be differently expressed and to regulate different cell types and pathways during cardiac aging [100, 101]. It has been showed that cardiac miR-21 is upregulated with age and induces a profibrotic effect *via* ERK–microtubule-associated protein (MAP) kinase pathway activation in cardiac fibroblasts (CFs) during aging (Table 1) [102].

Another miRNA involved in cardiac aging is miR-22. Age-related miR-22 upregulation contributed to accelerate CFs senescence and increased migration [103]. The effects of miR-22 in CFs were mediated by the proteoglycan mimecan/osteoglycin [103]. Interestingly, Mimecan/osteoglycin has been shown to regulate arteriogenesis, collagen fibrillogenesis in the ECM and cardiac hypertrophy [104]. Moreover, Gupta *et al.* [105] have recently suggested a role for miR-22 as an abundant and strong inhibitor of the cardiac autophagy process in aged cardiomyocytes. The inhibition of miR-22 stimulated cardiac autophagy, maladaptive remodeling and enhanced cardiac function post-MI in older mice, but not in younger ones [106]. miR-17-92 cluster has been widely shown to be involved in cardiac aging and recruits six mature miRNAs: miR-17, miR-18a, miR-19a, miR-19b, miR-20a, and miR-92a. It has been found that miR-18 and miR-19 levels were decreased in an aged HF-prone mouse strain. These findings were also confirmed in human samples, and this is crucial since HF is a frequent comorbidity in elderly patients. Indeed these authors have shown that expression of miR-17–92 cluster changes with cardiac aging and associates with decreased miR-18a, miR-19a, and miR-19b expression in age-related remodeling in the heart [46].

miR-34a expression was significantly augmented in aged human hearts, in a mouse model of accelerated aging or post-MI [107]. The inhibition of miR-34a through gene deletion or antagomiR was able to reverse both postischemic and age-related cardiac dysfunction.

### Circulating miRNAs in CVDs during aging

miRNAs are secreted in the extracellular space and are emerging as important molecules for paracrine and systemic communication between different cells, organs and systems.

Thus, a combination of specific circulating miRNAs would be a valuable tool to estimate the age-related deterioration of different organs. Currently, geriatricians are using numerous clinical multidimensional indexes to identify frail elderly patients and to predict the risk of mortality [108]. Therefore, adding new circulating biomarkers like circulating miRNAs to the current geriatric ones would be definitely valuable. Circulating miRNAs have potential uses for the diagnosis and prognosis of many age-related CVDs; their levels have been related with different ages and some of them have been associated with the so called successful aging. At this regard, it has been shown that aging influences circulating levels of some miRNAs in animal models of physiological aging as well as in human elderly subjects when compared to younger [109]. Analysis of circulating miRNAs found that miR-34b/c and miR-449 expression levels correlates with age [109]. This finding is not surprising since miR-34 family members (miR-34a, -34b, and -34c) have already been associated with cardiac dysfunction as well as Alzheimer's disease both in animal models and humans [110]. Moreover, it has been shown that plasma levels of miR-146a, already associated with endothelial senescence, rise in aged normal mice, but are unchanged in dwarf mice that have increased life span [109, 111]. Interestingly, cardiac miR-21 has been also shown to be increased in a mouse model of aging [100]. However, increased levels of circulating miR-21 in aged patients would be almost partially due to its connection with MI/age-related cardiac dysfunction and cancer, frequent comorbidities in elderly [100, 112]. Remarkably, miR-181 was downregulated in peripheral blood of older subject and further reduced in age-matched patients with HF [46].

## DOES PHYSICAL ACTIVITY MODIFY THE LEVELS OF CIRCULATING MIRNA DETECTED IN CVDS?

**Figure 1:**
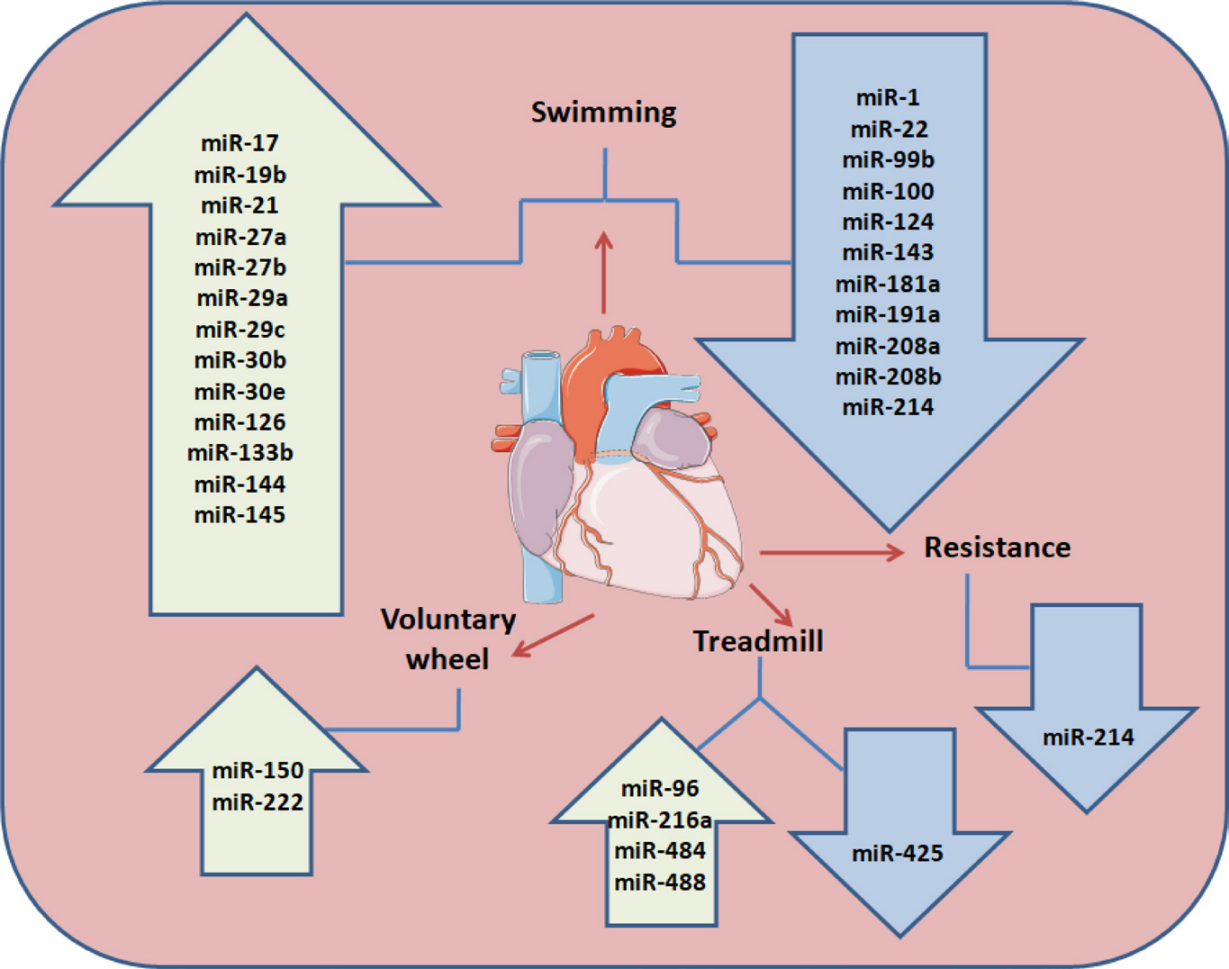
Exercise training regulation on animal model cardiac miRNAs Different training modalities (swimming exercise, resistance training, aerobic exercise training on motorized treadmill and voluntary running wheel) performed in animal models (mice and rats) up-or down regulate miRNAs expression in the blood stream.

Aerobic exercise training leads to a physiological, non-pathological LVH. However, in order to study the beneficial effects of exercise training (ET), a number of experimental strategies have been established, such as treadmill [26] and voluntary wheel running [113], swimming [113, 114] and resistance training (RT) (Figure 1) [115, 116].

These approaches have been applied to numerous animal models with various backgrounds. Most of the studies performed in rats and mice have been applied to continuous treadmill running, characterized by fixed or progressively increasing speed, inclination and duration during the session.

Additionally, swimming is recognized for its efficiency in inducing myocardial hypertrophy and a significant increase in LV end-diastolic volume in rats [114]. Furthermore it has been demonstrated that swimming exercise-induces physiological cardiac hypertrophy related with the modulation of some miRNAs (rno-miR-21, rno-miR-124, rno-miR-144, and rno-miR-145) that target components of the PI3K/AKT/mTOR signaling pathway (Pik3a, Pten and Tsc2) [117]. The studies described above collectively suggest that swimming ET in experimental animal models changes the cardiac levels of several miRNAs (miR-27a, -27b, -143, -29ac, and -126) (Figure 1) influencing a broad spectrum of targets, that are components of the RAS and Raf-1/Erk1/2 pathways and associates with different phenotypes, tissue remodeling and angiogenesis.

During the last few years a number of studies on circulating miRNAs in the bloodstream induced by both acute and chronic exercise have rised (Table 2) [118, 119]. Release of miRNAs into the circulation after acute exercise is likely to be a result of exercise-induced tissue stress and/or damage. For instance, Baggish and colleagues [120] reported that vascular endothelial cells are capable of releasing high levels of both miR-21 and miR-222 into the circulation immediately after acute exhaustive cycling exercise [121] (Table 2). As reported above, it has been demonstrated that miR-21 [112] and miR-222 are modified in MI and CAD pathological conditions [31].

**Table 2:** Circulating miRNA expression changes induced by acute and chronic exercise

Circulating miRNA (plasma) upregulated	Circulating miRNA (plasma) downregulated	Type of exercise	References
miR-9, miR-133a, miR-133b, miR-139, miR-143, miR-181b, miR-206, miR-208b, miR-214, miR-223, miR-330, miR-338, miR-485, miR-509, miR-517a, miR-518f, miR-520f, miR-522, miR-553, miR-888	Let-7i, miR-30b, miR-106a, miR-146a, miR-151, miR-221, miR-652	Acute exercise	Bye *et al.*, 2013; Baggish *et al.*, 2011; Nielsen *et al.* 2014;
miR-21, miR-146a, miR-221, miR-222		Acute and Chronic exercise	Aoi *et al.*, 2013; Baggish *et al.*, 2014; Bye *et al.*, 2013; Nielsen *et al.*, 2014; Taurino *et al.*, 2010
miR-20a, miR-103, miR-107, miR-126, miR-376a	let-7d, miR-16, miR-21, miR-25, miR-27a, miR-28, miR-148a, miR-185, miR-342, miR-766	Chronic exercise	Nielsen *et al.* 2014; Aoi *et al.*, 2013;

The table summarize circulating miRNA (plasma) up-or down-regulated after acute or chronic exercise.

These authors highlighted the rapid mobilization of circulating miRNAs in response to a cardiopulmonary exercise test, as they reported in the setting of acute exercise a rapid upregulation of miR-146a, miR-222, miR-21 and miR-221, followed by a return to the initial plasma levels after 1 h of rest in three of these circulating miRNAs (miR-146a, miR-222 and miR-21). As reported by Caruso *et al.* [54], miR-221 is modified in CAD.

Nielsen *et al.* [118] performed a screening for circulating miRNAs in young healthy men before and after an acute endurance exercise bout (60 min cycle ergometer exercise bout at 65% of maximal power (*P*_max_)) or following endurance exercise training (60–120 min/section on cycle ergometer, 5 times/wk, 12 wks). These authors demonstrated that exercise training bout induces a dynamic regulation of miRNAs in the circulation. For instance, immediately after the acute exercise bout, miR-106a, miR-221, miR-30b, miR-151-5p, let-7i, miR-146, miR-652 and miR-151-3p were significantly downregulated in the circulation. They also have showed that chronic exercise training induces changes in a completely different set of miRNAs (such as miR-139, miR-143, miR-223 and miR-330) in the circulation compared to an acute bout. It has been shown that miR-30 level was modified in hypertension [31]. One hour after the acute bout, 5 miRNAs (miR-338-3p, miR-330-3p, miR-223, miR-139-5p and miR-143) were found upregulated and after three hours only miR-1 remained increased. Likewise miR-139-5p was downregulated in HF, as well miR-143 levels in CH and also miR-223 levels were found in AF.

A few studies have also reported changes in circulating miRNAs after long-term exercise training protocols. Baggish *et al.* [120] studied subjects that performed moderate-intensity endurance exercise training (rowing) for 3 months and reported the increase of basal levels of miR-21, miR-146a, miR-221 and miR-222. These results suggest that this increase reflects a training response to exercise.

Uhlemann *et al.* [122] showed that the manner of exercise training is important for the miRNA-signature in the bloodstream. Whereas different protocols of endurance exercise training led to an acute increase in the endothelial-specific miR-126, resistance training caused an increase in the skeletal muscle-specific miR-133. About miR-133, it has been found modified in cardiovascular pathologies such as CAD and MI and miR-126 was found downregulated in stroke and hypertension and modified in MI and CHF. In another study, miRNAs levels were measured in serum samples from twelve healthy males performing a single resistance exercise session (bench press and leg press), consisting of five sets of 10 repetitions at 70% of maximum strength, with a 1 min rest between each set. Three days after the exercise session the authors found increased levels of miR-149 and decreased levels of miR-146a and miR-221, suggesting that miRNA levels change in response to acute resistance exercise, and miRNAs may play important roles in resistance-exercise-induced adaptation (Table 2). At variance, Aoi and colleagues [123] found that miR-486 levels were significantly decreased following both acute and chronic exercise (Table 2).

Circulating miRNAs have also been the subject of study in marathon athletes [119, 124]. Fourteen male marathon runners were investigated for myomiRNAs and inflammation related miRNAs in the plasma before, immediately after and 24h after a marathon run [124]. It was found that miR-1, miR-133a, and miR-206 correlated to aerobic performance parameters, such as maximal oxygen uptake (VO_2 max_). Interestingly, miR-1 showed a moderate negative correlation with fractional shortening (the fraction of any diastolic dimension that is lost in systole) (whereas miR-133a was positively correlated with the thickness of intraventricular septum). Baggish and colleagues [119] evaluated the levels of miRNA in muscle (miR-1, miR-133a, miR-499–5p), cardiac tissue (miR-208a) and the vascular endothelium (miR-126), as well as those related to inflammation (miR-146a) in male marathon runners at rest, immediately after and 24 h after the race. All the measured miRNAs were found modified after the marathon, in particular miR-1, miR-133a, miR-208a and miR-146a.

More recently, it has been reported from the Munich Marathon Study that both elite and non-elite runners exhibited increased plasma levels of miR-30a immediately after a marathon. Interestingly, miR-1 and miR-133a plasma levels also increased but showed significant changes only in elite runners [77]. Plasma levels in elite runner correlate with left atrial diameter suggesting that circulating miRNAs could potentially serve as biomarkers of atrial remodeling in athletes [77].

A recent study by Bye *et al.* [125] reported that circulating levels of miR-210 and miR-222 were significantly higher in individuals with low VO_2 max_ when compared to individuals with high VO_2 max_ during exercise. Another study Baggish and colleagues [120] reported a positive correlation between the circulating levels of miR-146a and VO_2 max_ in 10 male athletic subjects.

Studies performed showed that ET decreases cardiac miR-208a expression in both healthy Wistar and obese Zucker rats, induces upregulation of targets as thyroid hormone receptor associated protein 1 (THRAP-1), Purβ and Sox6 and improves the balance between the cardiac α and β myosin heavy chain (α-βMHC) gene expression [126]. miRNA-208a downregulation is involved in the increase in several targets that constitute a gene program to improve the contractile efficiency of the heart [127].

On the contrary miR-208 expression levels, they were found upregulated in CAD [128].

Another study by Taurino and colleagues [129] reported that long-term endurance exercise training (10 weeks) caused an increase in both miR-92a and miR-92b circulating levels in patients with CAD and a decrease in hypertension suggesting that miRNA expression profiling in whole blood is a promising tool.

## THE POTENTIAL ROLE OF MIRNAS AS BIOMARKERS FOR CVDS

Single miRNAs have multiple targets and the modulation of single miRNA can influence an entire gene network [127]. In 2008 for the first time it was independently demonstrated by different groups [130, 131] that miRNAs could be detected in blood and are also remarkably present in other body fluids like urine, cerebrospinal fluid, and saliva [132].

Several studies have since then shown that miRNAs can be secreted into the bloodstream by different organs including heart, vascular endothelial cells, skeletal muscle, liver and brain [123]. Evidence suggested that miRNAs are packaged in microparticles (exosomes, microvesicles, and apoptotic bodies) [133] or associated with RNA-binding proteins (e.g., Argonaute 2) [134] or lipoprotein complexes (high-density lipoprotein) [135]. Some circulating miRNAs might just be products of ongoing physiological or pathological processes in tissue.

Furthermore there is evidence indicating that specific circulating miRNAs may have important functions as intercellular communicators [136]. For instance, miRNA communication has been demonstrated between endothelial cells and endothelial apoptotic bodies, as well as between smooth muscle cells and cardiomyocytes [137, 138].

The discovery of miRNAs as promising new biomarkers in the field of CVD and CAD in particular has ignited great expectations. Interestingly, several studies have reported the association of changes in circulating miRNAs levels with particular physiological states such as with aging, physical activity, and pregnancy [139, 140]. miRNAs have emerged as promising clinical biomarkers of diseases [141]. It has been performed a prospective nested case-control study, in a 10-year observation period, and identified many circulating miRNAs (let-7d-5p, let-7g-5p, miR-26a-5p, miR-29c-3p, miR-103a-3p, miR-106a-5p, miR-148b-3p, miR-151a-5p, miR-424-5p, miR-660-5p) that predict future fatal acute MI in healthy participants to the Nord-Trøndelag Health (HUNT) Study [77, 142].

miRNA quantification showed organ- and cell-specific expression patterns of certain miRNAs in health condition [143] and *in vitro* findings suggest groups of miRNAs being specifically up or down regulated in different CVDs (CAD and MI) such as miR-1, miR-122, miR-126, miR-133a, miR-133b, miR-208a and miR-499 [128]. In hearts of patients who died by MI, miR-1 was upregulated in myocardium as compared to infarcted tissue or healthy adult hearts [144]. miR-21 is upregulated in cardiomyocytes shortly after initiation of ischemia whereas before cell death its concentration decreases [145]. Levels of miR-126 were up regulated in the non-infarcted areas after induced MI in rat hearts [146]. miR-208 and miR-133 levels are upregulated in CAD.

Stability in the circulation, tissue- and pathology-specific regulation as well as high sensitivity and specificity suggest that miRNAs' applicability as biomarkers for cardiovascular disease might even overcome protein-based biomarkers [147, 148].

The assessment of CVD risk supported by novel circulating biomarkers, such as miRNAs, is important to stratify individuals at high-risk, to optimize treatment strategies and to enhance our understanding of the underlying biology.

## CONCLUSIONS

Emerging evidence suggests that miRNAs are likely to be important in the heart's response to the physiological stress of exercise. Given the well-recognized cardiovascular benefits of exercise, elucidating the contribution of miRNAs to this response has the potential not only to reveal novel aspects of cardiovascular biology but also to identify new targets for therapeutic intervention that may complement those discovered through studies of cardiovascular diseases.

Aging is a complex phenomenon involving different systems and usually associated with several comorbidities. Indeed, we have described and summarized the main and specific miRNAs involved or altered in both cardiovascular and age-related pathologies.

The summarized results demonstrate that numerous miRNAs are released into circulation during and after the exercise and reflect the acute response to physiological stimuli. Moreover, the circulating miRNA expression pattern seems to be sensitive and specific for the type and intensity of exercise.

The application of circulating miRNAs as biomarkers represents a potential additional scheme in disease diagnosis and prognosis complementary to established protein-based biomarkers.

## References

[1] Go AS, Mozaffarian D, Roger VL, Benjamin EJ, Berry JD, Borden WB, Bravata DM, Dai S, Ford ES, Fox CS, Franco S, Fullerton HJ, Gillespie C (2013). Heart disease and stroke statistics—2013 update: a report from the American Heart Association. Circulation.

[2] Held C, Iqbal R, Lear SA, Rosengren A, Islam S, Mathew J, Yusuf S (2012). Physical activity levels, ownership of goods promoting sedentary behaviour and risk of myocardial infarction: results of the INTERHEART study. Eur Heart J.

[3] North BJ, Sinclair DA (2012). The intersection between aging and cardiovascular disease. Circ Res.

[4] Lakatta EG, Levy D (2003). Arterial and cardiac aging: major shareholders in cardiovascular disease enterprises: Part II: the aging heart in health: links to heart disease. Circulation.

[5] Guha K, McDonagh T (2013). Heart failure epidemiology: European perspective. Curr Cardiol Rev.

[6] Pescatello LS, Franklin BA, Fagard R, Farquhar WB, Kelley GA, Ray CA, American College of Sports Medicine (2004). American College of Sports Medicine position stand. Exercise and hypertension. Med Sci Sports Exerc.

[7] Hagberg JM, Park JJ, Brown MD (2000). The role of exercise training in the treatment of hypertension: an update. Sports Med.

[8] Warburton DE, Nicol CW, Bredin SS (2006). Prescribing exercise as preventive therapy. CMAJ.

[9] Shiroma EJ, Lee IM (2010). Physical activity and cardiovascular health: lessons learned from epidemiological studies across age, gender, and race/ethnicity. Circulation.

[10] Byberg L, Melhus H, Gedeborg R, Sundstrom J, Ahlbom A, Zethelius B, Berglund LG, Wolk A, Michaelsson K (2009). Total mortality after changes in leisure time physical activity in 50 year old men: 35 year follow-up of population based cohort. BMJ.

[11] Lakatta EG (2002). Age-associated cardiovascular changes in health: impact on cardiovascular disease in older persons. Heart Fail Rev.

[12] Chomistek AK, Chasman DI, Cook NR, Rimm EB, Lee IM (2013). Physical activity, genes for physical fitness, and risk of coronary heart disease. Med Sci Sports Exerc.

[13] Mampuya WM (2012). Cardiac rehabilitation past, present and future: an overview. Cardiovasc Diagn Ther.

[14] Myers J (2003). Cardiology patient pages. Exercise and cardiovascular health. Circulation.

[15] Landsberg L, Aronne LJ, Beilin LJ, Burke V, Igel LI, Lloyd-Jones D, Sowers J (2013). Obesity-related hypertension: pathogenesis, cardiovascular risk, and treatment: a position paper of The Obesity Society and the American Society of Hypertension. J Clin Hypertens (Greenwich).

[16] Myers J, Wagner D, Schertler T, Beer M, Luchinger R, Klein M, Rickli H, Muller P, Mayer K, Schwitter J, Dubach P (2002). Effects of exercise training on left ventricular volumes and function in patients with nonischemic cardiomyopathy: application of magnetic resonance myocardial tagging. Am Heart J.

[17] Kavanagh T, Mertens DJ, Shephard RJ, Beyene J, Kennedy J, Campbell R, Sawyer P, Yacoub M (2003). Long-term cardiorespiratory results of exercise training following cardiac transplantation. Am J Cardiol.

[18] Yusuf S, Hawken S, Ounpuu S, Dans T, Avezum A, Lanas F, McQueen M, Budaj A, Pais P, Varigos J, Lisheng L, Investigators IS (2004). Effect of potentially modifiable risk factors associated with myocardial infarction in 52 countries (the INTERHEART study): case-control study. Lancet.

[19] Mora S, Cook N, Buring JE, Ridker PM, Lee IM (2007). Physical activity and reduced risk of cardiovascular events: potential mediating mechanisms. Circulation.

[20] Hatle H, Stobakk PK, Molmen HE, Bronstad E, Tjonna AE, Steinshamn S, Skogvoll E, Wisloff U, Ingul CB, Rognmo O (2014). Effect of 24 sessions of high-intensity aerobic interval training carried out at either high or moderate frequency, a randomized trial. PLoS One.

[21] Weston KS, Wisloff U, Coombes JS (2014). High-intensity interval training in patients with lifestyle-induced cardiometabolic disease: a systematic review and meta-analysis. Br J Sports Med.

[22] Bouchard C, Peacock ZS, Troulis MJ (2016). Pediatric Vascular Tumors of the Head and Neck. Oral Maxillofac Surg Clin North Am.

[23] Leon AR, Abraham WT, Curtis AB, Daubert JP, Fisher WG, Gurley J, Hayes DL, Lieberman R, Petersen-Stejskal S, Wheelan K, Program MS (2005). Safety of transvenous cardiac resynchronization system implantation in patients with chronic heart failure: combined results of over 2,000 patients from a multicenter study program. J Am Coll Cardiol.

[24] Ferguson S, Gledhill N, Jamnik VK, Wiebe C, Payne N (2001). Cardiac performance in endurance-trained and moderately active young women. Med Sci Sports Exerc.

[25] Wisloff U, Najjar SM, Ellingsen O, Haram PM, Swoap S, Al-Share Q, Fernstrom M, Rezaei K, Lee SJ, Koch LG, Britton SL (2005). Cardiovascular risk factors emerge after artificial selection for low aerobic capacity. Science.

[26] Wisloff U, Loennechen JP, Currie S, Smith GL, Ellingsen O (2002). Aerobic exercise reduces cardiomyocyte hypertrophy and increases contractility, Ca2+ sensitivity and SERCA-2 in rat after myocardial infarction. Cardiovasc Res.

[27] Respress JL, van Oort RJ, Li N, Rolim N, Dixit SS, deAlmeida A, Voigt N, Lawrence WS, Skapura DG, Skardal K, Wisloff U, Wieland T, Ai X (2012). Role of RyR2 phosphorylation at S2814 during heart failure progression. Circ Res.

[28] Kemi OJ, Ellingsen O, Smith GL, Wisloff U (2008). Exercise-induced changes in calcium handling in left ventricular cardiomyocytes. Front Biosci.

[29] Small EM, Olson EN (2011). Pervasive roles of microRNAs in cardiovascular biology. Nature.

[30] Liu N, Olson EN (2010). MicroRNA regulatory networks in cardiovascular development. Dev Cell.

[31] Jamaluddin MS, Weakley SM, Zhang L, Kougias P, Lin PH, Yao Q, Chen C (2011). miRNAs: roles and clinical applications in vascular disease. Expert Rev Mol Diagn.

[32] Skommer J, Rana I, Marques FZ, Zhu W, Du Z, Charchar FJ (2014). Small molecules, big effects: the role of microRNAs in regulation of cardiomyocyte death. Cell Death Dis.

[33] Fernandes T, Magalhaes FC, Roque FR, Phillips MI, Oliveira EM (2012). Exercise training prevents the microvascular rarefaction in hypertension balancing angiogenic and apoptotic factors: role of microRNAs-16, -21, and -126. Hypertension.

[34] Dirkx E, da Costa Martins PA, De Windt LJ (2013). Regulation of fetal gene expression in heart failure. Biochim Biophys Acta.

[35] el Azzouzi H, Leptidis S, Dirkx E, Hoeks J, van Bree B, Brand K, McClellan EA, Poels E, Sluimer JC, van den Hoogenhof MM, Armand AS, Yin X, Langley S (2013). The hypoxia-inducible microRNA cluster miR-199a approximately 214 targets myocardial PPARdelta and impairs mitochondrial fatty acid oxidation. Cell Metab.

[36] van Rooij E, Sutherland LB, Thatcher JE, DiMaio JM, Naseem RH, Marshall WS, Hill JA, Olson EN (2008). Dysregulation of microRNAs after myocardial infarction reveals a role of miR-29 in cardiac fibrosis. Proc Natl Acad Sci USA.

[37] Li L, Cong Y, Gao X, Wang Y, Lin P (2017). Differential expression profiles of long non-coding RNAs as potential biomarkers for the early diagnosis of acute myocardial infarction. Oncotarget.

[38] Leptidis S, El Azzouzi H, Lok SI, de Weger R, Olieslagers S, Kisters N, Silva GJ, Heymans S, Cuppen E, Berezikov E, De Windt LJ, da Costa Martins P (2013). A deep sequencing approach to uncover the miRNOME in the human heart. PLoS One.

[39] Ikeda S, Kong SW, Lu J, Bisping E, Zhang H, Allen PD, Golub TR, Pieske B, Pu WT (2007). Altered microRNA expression in human heart disease. Physiol Genomics.

[40] van Oort RJ, van Rooij E, Bourajjaj M, Schimmel J, Jansen MA, van der Nagel R, Doevendans PA, Schneider MD, van Echteld CJ, De Windt LJ (2006). MEF2 activates a genetic program promoting chamber dilation and contractile dysfunction in calcineurin-induced heart failure. Circulation.

[41] Pang L, Hu J, Zhang G, Li X, Zhang X, Yu F, Lan Y, Xu J, Pang B, Han D, Xiao Y, Li X (2016). Dysregulated long intergenic non-coding RNA modules contribute to heart failure. Oncotarget.

[42] Kontaraki JE, Marketou ME, Zacharis EA, Parthenakis FI, Vardas PE (2014). MicroRNA-9 and microRNA-126 expression levels in patients with essential hypertension: potential markers of target-organ damage. J Am Soc Hypertens.

[43] Ali SS, Kala C, Abid M, Ahmad N, Sharma US, Khan NA (2016). Pathological microRNAs in acute cardiovascular diseases and microRNA therapeutics. Journal of Acute Disease.

[44] Da Costa Martins PA, De Windt LJ (2012). MicroRNAs in control of cardiac hypertrophy. Cardiovasc Res.

[45] Chopra S, Baby C, Jacob JJ (2011). Neuro-endocrine regulation of blood pressure. Indian J Endocrinol Metab.

[46] de Lucia C, Komici K, Borghetti G, Femminella GD, Bencivenga L, Cannavo A, Corbi G, Ferrara N, Houser SR, Koch WJ, Rengo G (2017). microRNA in Cardiovascular Aging and Age-Related Cardiovascular Diseases. Front Med (Lausanne).

[47] Mayet J, Hughes A (2003). Cardiac and vascular pathophysiology in hypertension. Heart.

[48] Fernandes T, Hashimoto NY, Magalhaes FC, Fernandes FB, Casarini DE, Carmona AK, Krieger JE, Phillips MI, Oliveira EM (2011). Aerobic exercise training-induced left ventricular hypertrophy involves regulatory MicroRNAs, decreased angiotensin-converting enzyme-angiotensin ii, and synergistic regulation of angiotensin-converting enzyme 2-angiotensin (1-7). Hypertension.

[49] Kohlstedt K, Trouvain C, Boettger T, Shi L, Fisslthaler B, Fleming I (2013). AMP-activated protein kinase regulates endothelial cell angiotensin-converting enzyme expression via p53 and the post-transcriptional regulation of microRNA-143/145. Circ Res.

[50] Boettger T, Beetz N, Kostin S, Schneider J, Kruger M, Hein L, Braun T (2009). Acquisition of the contractile phenotype by murine arterial smooth muscle cells depends on the Mir143/145 gene cluster. J Clin Invest.

[51] Feihl F, Liaudet L, Waeber B, Levy BI (2006). Hypertension: a disease of the microcirculation?. Hypertension.

[52] Renna NF (2013). Oxidative stress, vascular remodeling, and vascular inflammation in hypertension. Int J Hypertens.

[53] Wu WH, Hu CP, Chen XP, Zhang WF, Li XW, Xiong XM, Li YJ (2011). MicroRNA-130a mediates proliferation of vascular smooth muscle cells in hypertension. Am J Hypertens.

[54] Caruso P, MacLean MR, Khanin R, McClure J, Soon E, Southgate M, MacDonald RA, Greig JA, Robertson KE, Masson R, Denby L, Dempsie Y, Long L (2010). Dynamic changes in lung microRNA profiles during the development of pulmonary hypertension due to chronic hypoxia and monocrotaline. Arterioscler Thromb Vasc Biol.

[55] Jones Buie JN, Goodwin AJ, Cook JA, Halushka PV, Fan H (2016). The role of miRNAs in cardiovascular disease risk factors. Atherosclerosis.

[56] Lagos-Quintana M, Rauhut R, Yalcin A, Meyer J, Lendeckel W, Tuschl T (2002). Identification of tissue-specific microRNAs from mouse. Curr Biol.

[57] Gao W, He HW, Wang ZM, Zhao H, Lian XQ, Wang YS, Zhu J, Yan JJ, Zhang DG, Yang ZJ, Wang LS (2012). Plasma levels of lipometabolism-related miR-122 and miR-370 are increased in patients with hyperlipidemia and associated with coronary artery disease. Lipids Health Dis.

[58] Elmen J, Lindow M, Silahtaroglu A, Bak M, Christensen M, Lind-Thomsen A, Hedtjarn M, Hansen JB, Hansen HF, Straarup EM, McCullagh K, Kearney P, Kauppinen S (2008). Antagonism of microRNA-122 in mice by systemically administered LNA-antimiR leads to up-regulation of a large set of predicted target mRNAs in the liver. Nucleic Acids Res.

[59] Esau C, Davis S, Murray SF, Yu XX, Pandey SK, Pear M, Watts L, Booten SL, Graham M, McKay R, Subramaniam A, Propp S, Lollo BA (2006). miR-122 regulation of lipid metabolism revealed by in vivo antisense targeting. Cell Metab.

[60] Tsai WC, Hsu SD, Hsu CS, Lai TC, Chen SJ, Shen R, Huang Y, Chen HC, Lee CH, Tsai TF, Hsu MT, Wu JC, Huang HD (2012). MicroRNA-122 plays a critical role in liver homeostasis and hepatocarcinogenesis. J Clin Invest.

[61] Hsu SH, Wang B, Kota J, Yu J, Costinean S, Kutay H, Yu L, Bai S, La Perle K, Chivukula RR, Mao H, Wei M, Clark KR (2012). Essential metabolic, anti-inflammatory, and anti-tumorigenic functions of miR-122 in liver. J Clin Invest.

[62] Rotllan N, Price N, Pati P, Goedeke L, Fernandez-Hernando C (2016). microRNAs in lipoprotein metabolism and cardiometabolic disorders. Atherosclerosis.

[63] Soh J, Iqbal J, Queiroz J, Fernandez-Hernando C, Hussain MM (2013). MicroRNA-30c reduces hyperlipidemia and atherosclerosis in mice by decreasing lipid synthesis and lipoprotein secretion. Nat Med.

[64] Rayner KJ, Esau CC, Hussain FN, McDaniel AL, Marshall SM, van Gils JM, Ray TD, Sheedy FJ, Goedeke L, Liu X, Khatsenko OG, Kaimal V, Lees CJ (2011). Inhibition of miR-33a/b in non-human primates raises plasma HDL and lowers VLDL triglycerides. Nature.

[65] Rayner KJ, Sheedy FJ, Esau CC, Hussain FN, Temel RE, Parathath S, van Gils JM, Rayner AJ, Chang AN, Suarez Y, Fernandez-Hernando C, Fisher EA, Moore KJ (2011). Antagonism of miR-33 in mice promotes reverse cholesterol transport and regression of atherosclerosis. J Clin Invest.

[66] Sahoo D, Trischuk TC, Chan T, Drover VA, Ho S, Chimini G, Agellon LB, Agnihotri R, Francis GA, Lehner R (2004). ABCA1-dependent lipid efflux to apolipoprotein A-I mediates HDL particle formation and decreases VLDL secretion from murine hepatocytes. J Lipid Res.

[67] Slagsvold KH, Rognmo O, Hoydal M, Wisloff U, Wahba A (2014). Remote ischemic preconditioning preserves mitochondrial function and influences myocardial microRNA expression in atrial myocardium during coronary bypass surgery. Circ Res.

[68] Contu R, Latronico MV, Condorelli G (2010). Circulating microRNAs as potential biomarkers of coronary artery disease: a promise to be fulfilled?. Circ Res.

[69] Fichtlscherer S, De Rosa S, Fox H, Schwietz T, Fischer A, Liebetrau C, Weber M, Hamm CW, Roxe T, Muller-Ardogan M, Bonauer A, Zeiher AM, Dimmeler S (2010). Circulating microRNAs in patients with coronary artery disease. Circ Res.

[70] Fleissner F, Jazbutyte V, Fiedler J, Gupta SK, Yin X, Xu Q, Galuppo P, Kneitz S, Mayr M, Ertl G, Bauersachs J, Thum T (2010). Short communication: asymmetric dimethylarginine impairs angiogenic progenitor cell function in patients with coronary artery disease through a microRNA-21-dependent mechanism. Circ Res.

[71] Goren Y, Kushnir M, Zafrir B, Tabak S, Lewis BS, Amir O (2012). Serum levels of microRNAs in patients with heart failure. Eur J Heart Fail.

[72] Cakmak HA, Barman HA, Ikitimur B, Coskunpinar E, Oltulu YM, Can G, Karadag B, Altay S, Vural VA (2013). The Assessment of Relationship between Dysregulated MicroRNAs and Left Ventricular Mass and Mass Index in Systolic Heart Failure. Journal of the American College of Cardiology.

[73] Cakmak HA, Coskunpinar E, Ikitimur B, Barman HA, Karadag B, Tiryakioglu NO, Kahraman K, Vural VA (2015). The prognostic value of circulating microRNAs in heart failure: preliminary results from a genome-wide expression study. J Cardiovasc Med (Hagerstown).

[74] Li H, Fan J, Yin Z, Wang F, Chen C, Wang DW (2016). Identification of cardiac-related circulating microRNA profile in human chronic heart failure. Oncotarget.

[75] Wang N, Zhou Z, Liao X, Zhang T (2009). Role of microRNAs in cardiac hypertrophy and heart failure. IUBMB Life.

[76] Koutsis G, Siasos G, Spengos K (2013). The emerging role of microRNA in stroke. Curr Top Med Chem.

[77] Silva GJJ, Bye A, El Azzouzi H, Wisloff U (2017). MicroRNAs as Important Regulators of Exercise Adaptation. Prog Cardiovasc Dis.

[78] Long G, Wang F, Li H, Yin Z, Sandip C, Lou Y, Wang Y, Chen C, Wang DW (2013). Circulating miR-30a, miR-126 and let-7b as biomarker for ischemic stroke in humans. BMC Neurol.

[79] Liu X, Xiong Y, Zhou Z, Niu G, Wang W, Xiao G, Lin M, Leung TW, Liu D, Liu W, Fan X, Yin Q, Zhu W (2013). China interventional stroke registry: rationale and study design. Cerebrovasc Dis.

[80] Harris TA, Yamakuchi M, Ferlito M, Mendell JT, Lowenstein CJ (2008). MicroRNA-126 regulates endothelial expression of vascular cell adhesion molecule 1. Proc Natl Acad Sci U S A.

[81] Rodriguez-Yanez M, Sobrino T, Arias S, Vazquez-Herrero F, Brea D, Blanco M, Leira R, Castellanos M, Serena J, Vivancos J, Davalos A, Castillo J (2011). Early biomarkers of clinical-diffusion mismatch in acute ischemic stroke. Stroke.

[82] Sepramaniam S, Tan JR, Tan KS, DeSilva DA, Tavintharan S, Woon FP, Wang CW, Yong FL, Karolina DS, Kaur P, Liu FJ, Lim KY, Armugam A (2014). Circulating microRNAs as biomarkers of acute stroke. Int J Mol Sci.

[83] Ballabio E, Armesto M, Breeze CE, Manterola L, Arestin M, Tramonti D, Hatton CS, Lawrie CH (2012). Bortezomib action in multiple myeloma: microRNA-mediated synergy (and miR-27a/CDK5 driven sensitivity)?. Blood Cancer J.

[84] Mitsios N, Pennucci R, Krupinski J, Sanfeliu C, Gaffney J, Kumar P, Kumar S, Juan-Babot O, Slevin M (2007). Expression of cyclin-dependent kinase 5 mRNA and protein in the human brain following acute ischemic stroke. Brain Pathol.

[85] Yang ZB, Zhang Z, Li TB, Lou Z, Li SY, Yang H, Yang J, Luo XJ, Peng J (2014). Up-regulation of brain-enriched miR-107 promotes excitatory neurotoxicity through down-regulation of glutamate transporter-1 expression following ischaemic stroke. Clin Sci (Lond).

[86] Chen X, Lin M, Wang W (2017). The progression in atrial fibrillation patients with COPD: a systematic review and meta-analysis. Oncotarget.

[87] Santulli G, Iaccarino G, De Luca N, Trimarco B, Condorelli G (2014). Atrial fibrillation and microRNAs. Front Physiol.

[88] Montgomery RL, van Rooij E (2011). Therapeutic advances in MicroRNA targeting. J Cardiovasc Pharmacol.

[89] Lu Y, Zhang Y, Wang N, Pan Z, Gao X, Zhang F, Zhang Y, Shan H, Luo X, Bai Y, Sun L, Song W, Xu C (2010). MicroRNA-328 contributes to adverse electrical remodeling in atrial fibrillation. Circulation.

[90] Wang J, Yang X (2012). The function of miRNA in cardiac hypertrophy. Cell Mol Life Sci.

[91] Zhao Y, Srivastava D (2007). A developmental view of microRNA function. Trends Biochem Sci.

[92] Sayed D, Hong C, Chen IY, Lypowy J, Abdellatif M (2007). MicroRNAs play an essential role in the development of cardiac hypertrophy. Circ Res.

[93] Care A, Catalucci D, Felicetti F, Bonci D, Addario A, Gallo P, Bang ML, Segnalini P, Gu Y, Dalton ND, Elia L, Latronico MV, Hoydal M (2007). MicroRNA-133 controls cardiac hypertrophy. Nat Med.

[94] Dong Q, Meng P, Wang T, Qin W, Qin W, Wang F, Yuan J, Chen Z, Yang A, Wang H (2010). MicroRNA let-7a inhibits proliferation of human prostate cancer cells in vitro and in vivo by targeting E2F2 and CCND2. PLoS One.

[95] Lew JK, Pearson JT, Schwenke DO, Katare R (2017). Exercise mediated protection of diabetic heart through modulation of microRNA mediated molecular pathways. Cardiovasc Diabetol.

[96] Bartos DC, Grandi E, Ripplinger CM (2015). Ion Channels in the Heart. Compr Physiol.

[97] Strait JB, Lakatta EG (2012). Aging-associated cardiovascular changes and their relationship to heart failure. Heart Fail Clin.

[98] Goldspink DA, Gadsby JR, Bellett G, Keynton J, Tyrrell BJ, Lund EK, Powell PP, Thomas P, Mogensen MM (2013). The microtubule end-binding protein EB2 is a central regulator of microtubule reorganisation in apico-basal epithelial differentiation. J Cell Sci.

[99] Martin-Fernandez B, Gredilla R (2016). Mitochondria and oxidative stress in heart aging. Age (Dordr).

[100] Elia L, Contu R, Quintavalle M, Varrone F, Chimenti C, Russo MA, Cimino V, De Marinis L, Frustaci A, Catalucci D, Condorelli G (2009). Reciprocal regulation of microRNA-1 and insulin-like growth factor-1 signal transduction cascade in cardiac and skeletal muscle in physiological and pathological conditions. Circulation.

[101] Dahan D, Ekman M, Larsson-Callerfelt AK, Turczynska K, Boettger T, Braun T, Sward K, Albinsson S (2014). Induction of angiotensin-converting enzyme after miR-143/145 deletion is critical for impaired smooth muscle contractility. Am J Physiol Cell Physiol.

[102] Zhou Z, Guo F, Yi L, Tang J, Dou Y, Huan J (2015). The p38/mitogen-activated protein kinase pathway is implicated in lipopolysaccharide-induced microtubule depolymerization via up-regulation of microtubule-associated protein 4 phosphorylation in human vascular endothelium. Surgery.

[103] Kumar S, Berriochoa Z, Jones AD, Fu Y (2014). Detecting abnormalities in choroidal vasculature in a mouse model of age-related macular degeneration by time-course indocyanine green angiography. J Vis Exp.

[104] Ikeda S, He A, Kong SW, Lu J, Bejar R, Bodyak N, Lee KH, Ma Q, Kang PM, Golub TR, Pu WT (2009). MicroRNA-1 negatively regulates expression of the hypertrophy-associated calmodulin and Mef2a genes. Mol Cell Biol.

[105] Gupta SK, Foinquinos A, Thum S, Remke J, Zimmer K, Bauters C, de Groote P, Boon RA, de Windt LJ, Preissl S, Hein L, Batkai S, Pinet F (2016). Preclinical Development of a MicroRNA-Based Therapy for Elderly Patients With Myocardial Infarction. J Am Coll Cardiol.

[106] Yang B, Lin H, Xiao J, Lu Y, Luo X, Li B, Zhang Y, Xu C, Bai Y, Wang H, Chen G, Wang Z (2007). The muscle-specific microRNA miR-1 regulates cardiac arrhythmogenic potential by targeting GJA1 and KCNJ2. Nat Med.

[107] Wang S, Li J, Li XJ (2008). [Morphological and functional characteristics of pancreatic islet beta cells in natural aging SD rats]. Sichuan Da Xue Xue Bao Yi Xue Ban.

[108] Widera C, Gupta SK, Lorenzen JM, Bang C, Bauersachs J, Bethmann K, Kempf T, Wollert KC, Thum T (2011). Diagnostic and prognostic impact of six circulating microRNAs in acute coronary syndrome. J Mol Cell Cardiol.

[109] Hsu A, Chen SJ, Chang YS, Chen HC, Chu PH (2014). Systemic approach to identify serum microRNAs as potential biomarkers for acute myocardial infarction. Biomed Res Int.

[110] Peng L, Ma W, Yi F, Yang YJ, Lin W, Chen H, Zhang X, Zhang LH, Zhang F, Du Q (2014). MicroRNA profiling in Chinese patients with primary Sjogren syndrome reveals elevated miRNA-181a in peripheral blood mononuclear cells. J Rheumatol.

[111] Long G, Wang F, Duan Q, Chen F, Yang S, Gong W, Wang Y, Chen C, Wang DW (2012). Human circulating microRNA-1 and microRNA-126 as potential novel indicators for acute myocardial infarction. Int J Biol Sci.

[112] Olivieri F, Antonicelli R, Spazzafumo L, Santini G, Rippo MR, Galeazzi R, Giovagnetti S, D'Alessandra Y, Marcheselli F, Capogrossi MC, Procopio AD (2014). Admission levels of circulating miR-499-5p and risk of death in elderly patients after acute non-ST elevation myocardial infarction. Int J Cardiol.

[113] Konhilas JP, Maass AH, Luckey SW, Stauffer BL, Olson EN, Leinwand LA (2004). Sex modifies exercise and cardiac adaptation in mice. Am J Physiol Heart Circ Physiol.

[114] Evangelista FS, Brum PC, Krieger JE (2003). Duration-controlled swimming exercise training induces cardiac hypertrophy in mice. Braz J Med Biol Res.

[115] Barauna VG, Batista ML, Costa Rosa LF, Casarini DE, Krieger JE, Oliveira EM (2005). Cardiovascular adaptations in rats submitted to a resistance-training model. Clin Exp Pharmacol Physiol.

[116] Mostarda CT, Rodrigues B, de Moraes OA, Moraes-Silva IC, Arruda PB, Cardoso R, Scapini KB, Dos Santos F, De Angelis K, Irigoyen MC (2014). Low intensity resistance training improves systolic function and cardiovascular autonomic control in diabetic rats. J Diabetes Complications.

[117] Roncarati R, Viviani Anselmi C, Losi MA, Papa L, Cavarretta E, Da Costa Martins P, Contaldi C, Saccani Jotti G, Franzone A, Galastri L, Latronico MV, Imbriaco M, Esposito G (2014). Circulating miR-29a, among other up-regulated microRNAs, is the only biomarker for both hypertrophy and fibrosis in patients with hypertrophic cardiomyopathy. J Am Coll Cardiol.

[118] Nielsen S, Akerstrom T, Rinnov A, Yfanti C, Scheele C, Pedersen BK, Laye MJ (2014). The miRNA plasma signature in response to acute aerobic exercise and endurance training. PLoS One.

[119] Baggish AL, Park J, Min PK, Isaacs S, Parker BA, Thompson PD, Troyanos C, D'Hemecourt P, Dyer S, Thiel M, Hale A, Chan SY (2014). Rapid upregulation and clearance of distinct circulating microRNAs after prolonged aerobic exercise. J Appl Physiol (1985).

[120] Baggish AL, Hale A, Weiner RB, Lewis GD, Systrom D, Wang F, Wang TJ, Chan SY (2011). Dynamic regulation of circulating microRNA during acute exhaustive exercise and sustained aerobic exercise training. J Physiol.

[121] Xu T, Zhou Q, Che L, Das S, Wang L, Jiang J, Li G, Xu J, Yao J, Wang H, Dai Y, Xiao J (2016). Circulating miR-21, miR-378, and miR-940 increase in response to an acute exhaustive exercise in chronic heart failure patients. Oncotarget.

[122] Uhlemann M, Mobius-Winkler S, Fikenzer S, Adam J, Redlich M, Mohlenkamp S, Hilberg T, Schuler GC, Adams V (2014). Circulating microRNA-126 increases after different forms of endurance exercise in healthy adults. Eur J Prev Cardiol.

[123] Aoi W, Ichikawa H, Mune K, Tanimura Y, Mizushima K, Naito Y, Yoshikawa T (2013). Muscle-enriched microRNA miR-486 decreases in circulation in response to exercise in young men. Front Physiol.

[124] Mooren FC, Viereck J, Kruger K, Thum T (2014). Circulating microRNAs as potential biomarkers of aerobic exercise capacity. Am J Physiol Heart Circ Physiol.

[125] Bye A, Rosjo H, Aspenes ST, Condorelli G, Omland T, Wisloff U (2013). Circulating microRNAs and aerobic fitness--the HUNT-Study. PLoS One.

[126] Neves VJ, Fernandes T, Roque FR, Soci UP, Melo SF, de Oliveira EM (2014). Exercise training in hypertension: Role of microRNAs. World J Cardiol.

[127] van Rooij E, Olson EN (2007). microRNAs put their signatures on the heart. Physiol Genomics.

[128] Creemers EE, Tijsen AJ, Pinto YM (2012). Circulating microRNAs: novel biomarkers and extracellular communicators in cardiovascular disease?. Circ Res.

[129] Taurino R, Pozzi P, Zanasi T (2010). Facile characterization of polymer fractions from waste electrical and electronic equipment (WEEE) for mechanical recycling. Waste Manag.

[130] Lawrie CH (2008). MicroRNA expression in lymphoid malignancies: new hope for diagnosis and therapy?. J Cell Mol Med.

[131] Mitchell PS, Parkin RK, Kroh EM, Fritz BR, Wyman SK, Pogosova-Agadjanyan EL, Peterson A, Noteboom J, O'Briant KC, Allen A, Lin DW, Urban N, Drescher CW (2008). Circulating microRNAs as stable blood-based markers for cancer detection. Proc Natl Acad Sci U S A.

[132] Weber JA, Baxter DH, Zhang S, Huang DY, Huang KH, Lee MJ, Galas DJ, Wang K (2010). The microRNA spectrum in 12 body fluids. Clin Chem.

[133] Hunter MP, Ismail N, Zhang X, Aguda BD, Lee EJ, Yu L, Xiao T, Schafer J, Lee ML, Schmittgen TD, Nana-Sinkam SP, Jarjoura D, Marsh CB (2008). Detection of microRNA expression in human peripheral blood microvesicles. PLoS One.

[134] Arroyo JD, Chevillet JR, Kroh EM, Ruf IK, Pritchard CC, Gibson DF, Mitchell PS, Bennett CF, Pogosova-Agadjanyan EL, Stirewalt DL, Tait JF, Tewari M (2011). Argonaute2 complexes carry a population of circulating microRNAs independent of vesicles in human plasma. Proc Natl Acad Sci U S A.

[135] Vickers KC, Palmisano BT, Shoucri BM, Shamburek RD, Remaley AT (2011). MicroRNAs are transported in plasma and delivered to recipient cells by high-density lipoproteins. Nat Cell Biol.

[136] Kosaka N, Iguchi H, Ochiya T (2010). Circulating microRNA in body fluid: a new potential biomarker for cancer diagnosis and prognosis. Cancer Sci.

[137] Halkein J, Tabruyn SP, Ricke-Hoch M, Haghikia A, Nguyen NQ, Scherr M, Castermans K, Malvaux L, Lambert V, Thiry M, Sliwa K, Noel A, Martial JA (2013). MicroRNA-146a is a therapeutic target and biomarker for peripartum cardiomyopathy. J Clin Invest.

[138] Hergenreider E, Heydt S, Treguer K, Boettger T, Horrevoets AJ, Zeiher AM, Scheffer MP, Frangakis AS, Yin X, Mayr M, Braun T, Urbich C, Boon RA (2012). Atheroprotective communication between endothelial cells and smooth muscle cells through miRNAs. Nat Cell Biol.

[139] Chim SS, Shing TK, Hung EC, Leung TY, Lau TK, Chiu RW, Lo YM (2008). Detection and characterization of placental microRNAs in maternal plasma. Clin Chem.

[140] Radom-Aizik S, Zaldivar F, Haddad F, Cooper DM (2013). Impact of brief exercise on peripheral blood NK cell gene and microRNA expression in young adults. J Appl Physiol (1985).

[141] Adachi T, Nakanishi M, Otsuka Y, Nishimura K, Hirokawa G, Goto Y, Nonogi H, Iwai N (2010). Plasma microRNA 499 as a biomarker of acute myocardial infarction. Clin Chem.

[142] Wang GK, Zhu JQ, Zhang JT, Li Q, Li Y, He J, Qin YW, Jing Q (2010). Circulating microRNA: a novel potential biomarker for early diagnosis of acute myocardial infarction in humans. Eur Heart J.

[143] van Rooij E (2011). The art of microRNA research. Circ Res.

[144] Bostjancic E, Zidar N, Stajner D, Glavac D (2010). MicroRNA miR-1 is up-regulated in remote myocardium in patients with myocardial infarction. Folia Biol (Praha).

[145] Ye Y, Perez-Polo JR, Qian J, Birnbaum Y (2011). The role of microRNA in modulating myocardial ischemia-reperfusion injury. Physiol Genomics.

[146] Dong S, Cheng Y, Yang J, Li J, Liu X, Wang X, Wang D, Krall TJ, Delphin ES, Zhang C (2009). MicroRNA expression signature and the role of microRNA-21 in the early phase of acute myocardial infarction. J Biol Chem.

[147] Paiva S, Agbulut O (2017). MiRroring the Multiple Potentials of MicroRNAs in Acute Myocardial Infarction. Front Cardiovasc Med.

[148] Vegter EL, Schmitter D, Hagemeijer Y, Ovchinnikova ES, van der Harst P, Teerlink JR, O'Connor CM, Metra M, Davison BA, Bloomfield D, Cotter G, Cleland JG, Givertz MM (2016). Use of biomarkers to establish potential role and function of circulating microRNAs in acute heart failure. Int J Cardiol.

